# Cardiometabolic characterization in metabolic dysfunction–associated fatty liver disease

**DOI:** 10.3389/fmed.2022.1023583

**Published:** 2022-10-20

**Authors:** Carolina M. Perdomo, Jorge M. Núñez-Córdoba, Ana Ezponda, Francisco J. Mendoza, Javier Ampuero, Gorka Bastarrika, Gema Frühbeck, Javier Escalada

**Affiliations:** ^1^Department of Endocrinology and Nutrition, Clínica Universidad de Navarra, Pamplona, Spain; ^2^Research Support Service, Central Clinical Trials Unit, Clínica Universidad de Navarra, Pamplona, Spain; ^3^Department of Radiology, Clínica Universidad de Navarra, Pamplona, Spain; ^4^Department of Gastroenterology, Hospital Universitario Virgen del Rocío, Instituto de Biomedicina de Sevilla, Universidad de Sevilla, Sevilla, Spain; ^5^Centro de Investigación Biomédica en Red de Enfermedades Hepáticas y Digestivas (CIBEREHD), Instituto de Salud Carlos III, Madrid, Spain; ^6^IdiSNA (Instituto de Investigación en la Salud de Navarra), Pamplona, Spain; ^7^Centro de Investigación Biomédica en Red de la Fisiopatología de la Obesidad y Nutrición (CIBEROBN), Instituto de Salud Carlos III, Madrid, Spain

**Keywords:** coronary artery calcium, epicardial adipose tissue, visceral adipose tissue, nonalcoholic fatty liver disease, metabolic dysfunction-associated fatty liver disease

## Abstract

**Background:**

To better understand the patient's heterogeneity in fatty liver disease (FLD), metabolic dysfunction–associated fatty liver disease (MAFLD) was proposed by international experts as a new nomenclature for nonalcoholic fatty liver disease (NAFLD). We aimed to evaluate the cardiovascular risk, assessed through coronary artery calcium (CAC) and epicardial adipose tissue (EAT), of patients without FLD and patients with FLD and its different subtypes.

**Methods:**

Cross sectional study of 370 patients. Patients with FLD were divided into 4 groups: FLD without metabolic dysfunction (non-MD FLD), MAFLD and the presence of overweight/obesity (MAFLD-OW), MAFLD and the presence of two metabolic abnormalities (MAFLD-MD) and MAFLD and the presence of T2D (MAFLD-T2D). MAFLD-OW included two subgroups: metabolically healthy obesity (MHO) and metabolically unhealthy obesity (MUHO). The patients without FLD were divided into 2 groups: patients without FLD and without MD (non-FLD nor MD; reference group) and patients without FLD but with MD (non-FLD with MD). EAT and CAC (measured through the Agatston Score) were determined by computed tomography.

**Results:**

Compared with the reference group (non-FLD nor MD), regarding EAT, patients with MAFLD-T2D and MAFLD-MUHO had the highest risk for CVD (OR 15.87, 95% CI 4.26-59.12 and OR 17.60, 95% CI 6.71-46.20, respectively), patients with MAFLD-MHO were also at risk for CVD (OR 3.62, 95% CI 1.83-7.16), and patients with non-MD FLD did not have a significantly increased risk (OR 1.77; 95% CI 0.67-4.73). Regarding CAC, patients with MAFLD-T2D had an increased risk for CVD (OR 6.56, 95% CI 2.18-19.76). Patients with MAFLD-MUHO, MAFLD-MHO and non-MD FLD did not have a significantly increased risk compared with the reference group (OR 2.54, 95% CI 0.90-7.13; OR 1.84, 95% CI 0.67-5.00 and OR 2.11, 95% CI 0.46-9.74, respectively).

**Conclusion:**

MAFLD–T2D and MAFLD–OW phenotypes had a significant risk for CVD. MAFLD new criteria reinforced the importance of identifying metabolic phenotypes in populations as it may help to identify patients with higher CVD risk and offer a personalized therapeutic management in a primary prevention setting.

## Original research

In 2020, metabolic dysfunction–associated fatty liver disease (MAFLD) was proposed by a panel of international experts as a new nomenclature for non-alcoholic fatty liver disease (NAFLD) considering the metabolic overload of each patient independently of the presence or not of other liver diseases (1). Experts worldwide pursued to better understand the patient's heterogeneity in fatty liver disease (FLD) and, therefore, help in patient stratification for management and prevention of disease progression (1, 2). Since then, different studies worldwide have evidenced that MAFLD criteria predicts mortality more effectively (3–5) and help to discriminate patients at high risk of disease progression (6), probably due to the fact that the MAFLD definition better identifies patients with significant fibrosis (3, 7, 8), and fibrosis is the major determinant of adverse outcomes (9).

The association between NAFLD and cardiovascular disease (CVD) has been broadly described in the literature (10). Evidence sustain that NAFLD should be considered as an independent risk factor for the development of CVD (11, 12). In comparison to NAFLD diagnosis criteria, MAFLD criteria may identify a greater number of patients with metabolic abnormalities (6–8, 13, 14), and consequently, an increased risk for heart alterations. Recent studies have assessed the association between MAFLD and the risk of CVD in a primary prevention setting (15), MAFLD definition better identify patients with worse CVD risk (analyzed through the Suita score) than the NAFLD criteria (15). Moreover, a significant increase in CVD was evidenced in a cohort of patients with MAFLD and concomitant viral infection compared to cases with MAFLD only (16). Furthermore, there is evidence that those who are excluded by the NAFLD definition but captured by the MAFLD definition are at higher cardiovascular risk than those excluded by the MAFLD definition but captured by the NAFLD definition (17). To date, the clinical impact of the change in nomenclature on the capacity to detect individuals at risk for CVD has not yet been clarified. We think that the MAFLD new criteria may help identify patients with high risk for CVD. Therefore, in this cross-sectional study, we aimed to describe the cardiovascular risk and subclinical cardiovascular disease, assessed through epicardial adipose tissue (EAT) and coronary artery calcium (CAC), of patients with FLD and its different subtypes.

## Patients and methods

### Patient population

**Figure 1:**
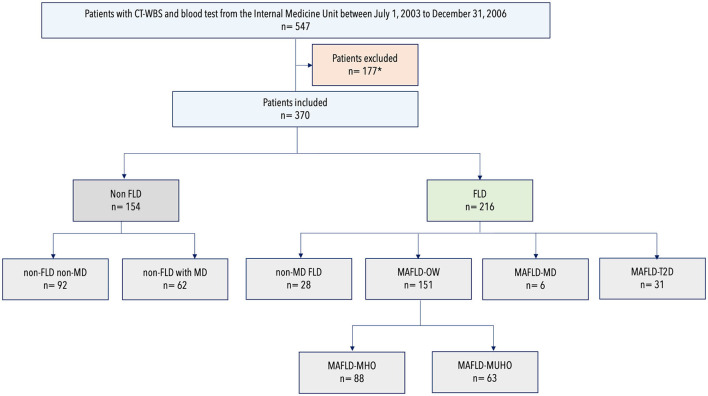
Flowchart of patient participation and classification according to metabolic dysfunction and fatty liver disease status. CT-WBS, Computed Tomography Whole Body Scan; FLD, fatty liver disease; MAFLD, metabolic dysfunction–associated fatty liver disease; MD, metabolic dysfunction; MHO, metabolically healthy obesity; MUHO, metabolically unhealthy obesity; OW, overweight; T2D, type 2 diabetes. *Of the initial cohort of 547 patients, 177 were excluded for one or more of the following criteria: personal history of cardiovascular disease (*n* = 50); personal history of non-ischemic heart disease (*n* = 25); personal history of cerebrovascular disease (*n* = 12); active malignancy (*n* = 14); excessive alcohol consumption (*n* = 32); advanced liver disease (*n* = 21) and unavailability to retrieve CT-WBS imaging (*n* = 3).

The study protocol was approved by the ethics committee of the Clínica Universidad de Navarra (Protocol Number 2019.080). In this retrospective study, we reviewed the records of subjects who underwent routine health checkups, had a computed tomography whole body scan (CT-WBS) and blood test in the same visit at Clínica Universidad de Navarra in Pamplona, Spain from July 1, 2003 to December 31, 2006. In our Center, CT-WBS and laboratory tests are routinely performed on the same day (or within a few days) of the initial visit. Exclusion criteria included ischemic heart diseases, heart failure, atrial fibrillation, pericarditis or valvular disease; personal history of cerebral vascular diseases (including transient ischemic attack); excessive alcohol consumption; advanced liver disease of other etiologies and malignant disease (Figure 1). Alcohol consumption was specifically investigated by interviewing each patient. Patients were classified into non-drinkers, moderate drinkers (average of less than one drink per day for women and less than two drinks per day for men) and heavy drinkers (average of one to less than three drinks per day for women, two to less than four drinks per day for men)(18). Excessive drinkers (average of three or more drinks per day for women, four or more drinks per day for men) were excluded from this cohort.

Extensive demographic, clinical (age, gender, smoking status, alcohol consumption, active medication list, personal and family medical history, anthropometrics), laboratory and radiological information were obtained from patient records. FLD was defined by evidence of hepatic steatosis on CT-WBS. MAFLD was defined as FLD in addition to one of the following three criteria: overweight/obesity, presence of T2D, or evidence of metabolic abnormalities. Body mass index (BMI) was calculated using the following formula: weight (in kilograms)/height (in meters^2^). Weight categories were classified as follows: normal weight (18.5–24.9 kg/m^2^), overweight (25.0–29.9 kg/m^2^), obesity class 1 (30.0–34.9 kg/m^2^), obesity class 2 (35.0–39.9 kg/m^2^) and obesity class 3 (≥40.0 kg/m^2^). The presence of metabolic dysregulation (MD) among normal weight individuals with FLD who did not have T2D was defined as the presence of two or more of the following metabolic abnormalities: (1) waist circumference ≥ 102 cm in men and 88 cm in women, (2) blood pressure ≥ 130/85 mmHg or specific drug treatment, (3) serum triglycerides (TG) ≥ 150 mg/dl (1.70 mmol/l) or specific drug treatment, (4) high-density lipoprotein (HDL) cholesterol <40 mg/dl (<1.0 mmol/l) for men and <50 mg/dl (<1.3 mmol/l) for women, (5) prediabetes (i.e., fasting glucose levels 100–125 mg/dl [5.6–6.9 mmol/l], or 2-h post-load glucose levels 140–199 mg/dl [7.8–11.0 mmol/l] or HbA1c 5.7–6.4%), (6) HOMA-IR score ≥ 2.5, and (7) a plasma C-reactive protein level >2 mg/L.

Patients with FLD were divided into 4 groups: FLD without metabolic dysfunction (non-MD FLD), MAFLD and the presence of overweight/obesity (MAFLD-OW), MAFLD and the presence of two metabolic abnormalities (MAFLD-MD) and MAFLD and the presence of T2D (MAFLD-T2D). MAFLD-OW included two subgroups: metabolically healthy obesity (MHO) and metabolically unhealthy obesity (MUHO). MUHO was defined as having overweight/obesity and at least two of the following cardiometabolic abnormalities: (1) blood pressure ≥ 130/85 mmHg or specific drug treatment, (2) serum TG ≥ 150 mg/dl (1.70 mmol/l) or specific drug treatment, (3) HDL cholesterol <40 mg/dl (<1.0 mmol/l) for men and <50 mg/dl (<1.3 mmol/l) for women, (4) prediabetes/diabetes (i.e., fasting glucose levels ≥ 100 [≥ 5.6], or 2-h post-load glucose levels ≥ 140 to 199 mg/dl [≥ 7.8] or HbA1c ≥ 5.7%) (19). The patients without FLD were divided into 2 groups to establish a reference group for logistic regression analysis: patients without FLD and without MD (non-FLD nor MD; reference group) and patients without FLD but with MD (non-FLD with MD).

### Non-invasive liver fibrosis serum marker

We used the BAAT Score (20) as the non-invasive fibrosis serum marker. The BAAT Score was calculated by the sum of the following variables: BMI≥28 kg/m^2^ (1 point), age ≥50 years (1 point), ALT ≥2 times the normal upper value (1 point), and TG ≥150 mg/dl (1.70 mmol/l) (1 point). A BAAT score ≤ 1 points is considered as low likelihood of liver fibrosis and a BAAT score ≥4 points have a high likelihood of liver fibrosis. A score between 2 and 3 points is considered as an indeterminant score.

### Cardiac function

Cardiac function was assessed through echocardiographic study (Sonos 5500, Hewlett-Packard, 3 MHz probe) which was performed in left lateral decubitus. Images were taken in the parasternal long- and short-axis views, two- and four- chamber apical views, and subxiphoid view.

### Whole-body scan computed tomography protocol

All CT-WBS were performed using a sixty-four-row multidetector CT (SOMATOM Definition and SOMATOM Sensation-64 from Siemens Healthcare; Forchheim, Germany). All images were stored in picture archiving and communication system (PACS). The protocol of CT-WBS includes low-dose chest CT (120 kV and 40 mA/s) without contrast material, CAC measurement through Agatston Score (120 kV and 138 mA/s), abdominopelvic CT (120 kV and 180 mA/s) performed after intravenous injection of 120-ml iodinated contrast medium at 2 ml/s (Omnipaque TM 300 [iohexol], 300 mg I/ml from GE Healthcare Bio-Sciences; Madrid, Spain); portal phase was acquired at 65 seconds. CAC through Agatston Score was categorized as 4 categories according to the degree of calcification (0: minimal risk; 0–99: mild risk; 100–399: moderate risk; >400: severe risk).

From January 2020 to December 2021, CT-WBS images were reobtained from PACS to measure EAT, visual scoring of CAC, liver and spleen attenuation, subcutaneous adipose tissue (SCAT) and visceral adipose tissue (VAT) by two radiologists, blinded to clinical data. EAT, VAT and SCAT were semiautomatically quantified in a research prototype software (Syngo.via Frontier-Cardiac risk assessment application; Siemens, AG; Healthcare Sector, Germany). EAT was defined as all cardiac adipose tissue, including the epi- and pericardial fat. EAT was semi-automatically quantified including voxels with attenuation values between−45 and−190 Hounsfield units (HU). Adjusting for body surface area, indexed epicardial adipose tissue (EATi) was also calculated; the upper normal limit of EATi was 68.1 cm^3^/m^2^ (21). The Du Bois method was used (0.20247 x height (m)^0.725^ x weight (kg)^0.425^) to calculate the body surface area (22). The overall abdominopelvic VAT and SCAT volumes were obtained with the attenuation-based method. The outer contour of the abdominal muscular wall was manually traced to differentiate VAT (inner) and SCAT (outer). On the longitudinal axis the analyzed region ranged from the upper abdomen (adrenal gland level) to the L5/S1 intervertebral disc. Default thresholds (−150 to 50 HU) obtained from the total volume were employed to semiautomatically quantified VAT and SCAT. The VAT/SCAT ratio was calculated due to its known correlation to cardiovascular risk, beyond BMI and VAT (23).

### Statistical analysis

Demographic and clinical characteristics of patients were summarized using mean and standard deviation (SD) for continuous variables and percentages for categorical variables. The Kolmogorov-Smirnov test was used to assess the normal distribution of quantitative variables. Multiple group comparisons were done by analysis of variance (ANOVA) for normally distributed data. We used the chi-square test or Fisher's exact test for categorical variables. We used the analysis of covariance (ANCOVA) to calculate age- and sex-adjusted means and 95% confidence intervals (95% CI). Correlations were evaluated with the estimation of the product-moment correlation coefficient (r). The logistic regression was used to estimate age- and sex-adjusted odds ratios (OR) and 95% confidence intervals (95% CI). All analyses were performed with Stata 14 (StataCorp. 2015. Stata Statistical Software: Release 14. College Station, TX: StataCorp LP). *P* <0.05 was considered statistically significant.

## Results

A total of 370 patients were included in the analysis: 154 without FLD and 216 with FLD. Mean age was 57.9 ± 9.2 years and 71.2% (263/370) of the cohort were men. Of the 154 patients without FLD, 40.3% (62/154) were patients with MD (non-FLD with MD) and 59.7% (92/154) were patients without MD (non-FLD nor MD). Of the FLD cohort: 13.0% (28/216) were patients with hepatic steatosis but without metabolic dysfunction (non-MD FLD), 69.9% (151/216) were patients with MAFLD due to the presence of overweight/obesity (MAFLD-OW), 2.8% (6/216) were patients with MAFLD due to the presence of two metabolic abnormalities (MAFLD-MD) and 14.4% (31/216) were patients with MAFLD due to the presence of T2D (MAFLD-T2D). Of the 151 patients with MAFLD-OW, 58.3% (88/151) were patients with MAFLD-MHO and 41.7% (63/151) were patients with MAFLD-MUHO.

### Clinical characteristics of the patients included in the study

Table 1 displays the main demographic, clinical and laboratory characteristics. Compared with patients without FLD, patients with FLD had increased glycemia, insulinemia, homeostasis model assessment for insulin resistance (HOMA-IR), a more detrimental lipid profile (atherogenic dyslipidemia), hyperuricemia and worse kidney and liver function (*p* < 0.05). Additionally, a higher prevalence in males with metabolic syndrome disorders (impaired fasting glucose/diabetes, dyslipidemia, hyperuricemia, overweightness, and obesity) was detected in patients with FLD (*p* < 0.05). Indices of adiposity (BMI, waist circumference, VAT, SCAT, VAT/SCAT ratio) were higher in participants with FLD compared with patients without FLD (*p* < 0.05). Cardiac function was assessed on 69 of the patients (Supplementary Table 1).

**Table 1:** Clinical characteristics of the participants according to fatty liver disease status.

	**All patients**	**Non-FLD**	**FLD**	***p*-value**
*n*	370	154	216	
Age, *y*	58.0 ± 9.2	57.8 ± 10.0	58.1 ± 8.7	0.782
Men, *n* (%)	263 (71.1)	92 (59.7)	171 (79.2)	<0.001
BMI, kg/m^2^	27.6 ± 4.0	26.4 ± 4.0	28.5 ± 3.8	<0.001
Waist circumf., cm	99 ± 12	95 ± 13	101 ± 10	0.066
CUNBAE, %	32.4 ± 5.8	32.3 ± 6.2	32.6 ± 5.5	0.646
Hypertension, *n* (%)	92 (24.9)	31 (20.1)	61 (28.2)	0.075
Prediabetes, *n* (%)	135 (36.5)	34 (22.1)	101 (46.8)	<0.001
Diabetes, *n* (%)	39 (10.5)	8 (5.2)	31 (14.4)	0.005
Dyslipidaemia, *n* (%)	113 (30.5)	34 (22.1)	79 (36.6)	0.003
Current Smoking, *n* (%)	157 (46.3)	63 (44.7)	94 (47.5)	0.439
Alcohol consumption, *n* (%)	136 (36.8)	51 (47.2)	85 (55.2)	0.204
CVD family history, *n* (%)	94 (31.9)	35 (28.2)	59 (34.5)	0.253
Antihypertensive therapy, *n* (%)	74 (20.0)	31 (20.1)	43 (19.9)	0.958
Lipid-lowering therapy, *n* (%)	41 (11.1)	19 (12.4)	22 (10.2)	0.501
Antiplatelet therapy, *n* (%)	23 (6.2)	9 (5.8)	14 (6.5)	0.802
Glucose (mg/dL)	104 ± 27	98 ± 18	108 ± 31	<0.001
Insulin (U/mL)	12.6 ± 8.0	9.9 ± 5.9	14.1 ± 8.7	0.007
HOMA-IR	3.5 ± 2.6	2.6 ± 1.8	4.0 ± 2.8	<0.005
Triacylglycerol (mg/dL)	116.6 ± 77.2	93 ± 59	132 ± 84	<0.001
Total cholesterol (mg/dL)	221.5 ± 40.8	214 ± 37	226 ± 43	0.005
LDL cholesterol (mg/dL)	144.4 ± 36.3	140 ± 30	148 ± 40	0.048
HDL cholesterol (mg/dL)	54.0 ± 15.6	56 ± 17	52 ± 14	0.015
Urate (mg/dl)	5.7 ± 1.5	5.2 ± 1.3	6.1 ± 1.5	<0.001
ALT, IU/L	20.3 ± 12.2	16 ± 8	23 ± 14	<0.001
ALP, IU/L	91.2 ± 28.8	91 ± 28	91 ± 29	0.993
GGT, IU/L	26.2 ± 27.0	21 ± 16	30 ± 32	0.003
Creatinine (mg/dl)	0.9 ± 0.2	0.9 ± 0.2	1.0 ± 0.2	<0.001
GFR MDRD (ml/min/1.73 m^2^)	79.8 ± 16.2	81.6 ± 17.9	78.6 ± 14.8	0.075
GFR CPK-EPI (ml/min/1.73 m^2^)	88.4 ± 15.1	89.7 ± 16.1	87.4 ± 14.2	0.169
Urine Albumin to creatinine ratio, mg/g	1.9 ± 2.8	1.6 ± 2.0	2.2 ± 3.1	0.142
BAAT Score	-	-	2 ± 1	-

Values are expressed as mean (SD), unless otherwise stated.

*ALT*, alanine aminotransferase; *ALP*, alkaline phosphatase; *BMI*, body mass index; *CUNBAE*, Clínica Universidad de Navarra-body adiposity estimator; *CPK-EPI*, chronic kidney disease epidemiology collaboration equation; *FLD*, fatty liver disease; *GGT*, glutamyl transferase; *GFR*, glomerular filtration rate; *HDL*, high density lipoprotein; *HOMA*, homeostasis model assessment for insulin resistance; *LDL*, low density lipoprotein; *MAFLD*, metabolic dysfunction–associated fatty liver disease; *MD*, metabolic dysfunction; *MDRD*, modification of diet in renal disease equation.

**Figure 2:**
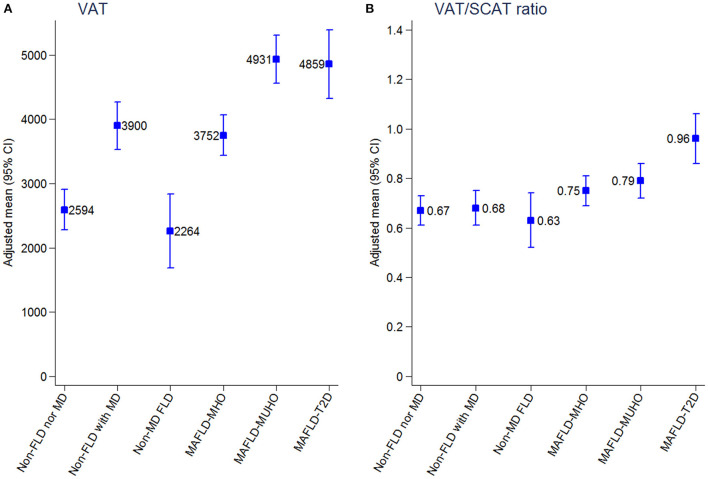
**(A,B)** Adjusted means of metabolic phenotypes of fatty liver disease with visceral adipose tissue and visceral adipose tissue/subcutaneous adipose tissue ratio. 95% CI, 95% confidence interval; FLD, fatty liver disease; MAFLD, metabolic dysfunction–associated fatty liver disease; MD, metabolic dysfunction; MHO, metabolically healthy obesity; MUHO, metabolically unhealthy obesity; T2D, type 2 diabetes; VAT, visceral adipose tissue; VAT/SCAT ratio, visceral adipose tissue/subcutaneous adipose tissue ratio.

Patients with MAFLD-T2D had worse serum concentrations of glucose, HOMA-IR, triglycerides, HDL-cholesterol, urine albumin to creatinine ratio, ALT and GGT (Table 2). A higher prevalence of dyslipidemia was detected in patients with MAFLD-MD. Patients with MAFLD-T2D had the highest average values of VAT, SCAT, VAT/SCAT ratio, BAAT Score, EAT, EATi, and CAC (Table 3). Figure 2 displays the age- and sex-adjusted means of VAT and VAT/SCAT ratio of the different metabolic phenotypes in our cohort (Supplementary Table 2).

**Table 2:** Clinical characteristics of the participants according to groups of metabolic dysfunction and fatty liver disease status.

	**Non-FLD nor MD**	**Non-FLD with MD**	**non-MD FLD**	**MAFLD-MHO**	**MAFLD-MUHO**	**MAFLD-MD**	**MAFLD-T2D**
*n*	92	62	28	88	63	6	31
Age, *y*	59.1 ± 11.0	55.9 ± 7.9	57.1 ± 9.4	57.5 ± 8.2	57.5 ± 8.6	60.5 11.4	61.6 ± 8.6
Men, *n* (%)	44 (47.8)	48 (77.4)	8 (28.6)	72 (81.8)	58(92.1)	4 (66.7)	29 (93.6)
BMI, kg/m^2^	24.6 ± 3.1	28.9 ± 3.7	23.2 ± 1.3	28.5 ± 2.8	30.4 ± 3.8	23.7 ± 1.2	30.4 ± 2.9
Waist circumf., cm	87 ± 6	103 ± 12	83 ± 5	97 ± 9	104 ± 10	84 ± 0	105 ± 6
CUNBAE, %	31.7 ± 6.1	33.1 ± 6.4	31.9 ± 6.3	32.2 ± 5.7	33.2 ± 5.2	28.5 ± 6.2	33.5 ± 4.9
Severe steatosis, *n* (%)	-	-	2 (7.1)	11 (12.5)	24 (38.1)	1 (16.7)	13 (41.9)
Hypertension, *n* (%)	7 (7.6)	26 (41.9)	2 (7.14)	16 (18.2)	25 (39.7)	3 (50)	15 (48.4)
Prediabetes, *n* (%)	4 (4.35)	22 (35.5)	5 (17.9)	18 (20.5)	49 (77.8)	3 (50)	0 (0)-
Dyslipidaemia, *n* (%)	7 (7.6)	27 (43.6)	3 (10.7)	21 (23.9)	33 (52.4)	5 (83.3)	17 (54.8)
Current Smoking, *n* (%)	35 (42.2)	28 (48.3)	15 (57.7)	32 (39.5)	29 (48.3)	3 (60)	15 (57.7)
Alcohol consumption, *n* (%)	27 (43.6)	24 (52.2)	10 (52.6)	32 (50.8)	32 (68.1)	1 (16.7)	10 (45.5)
Moderate alcohol consumption, *n* (%)	22 (23.9)	21 (33.9)	10 (35.7)	30 (34.1)	29 (46.0)	1 (16.7)	9 (29.0)
Heavy drinkers, *n* (%)	5 (5.4)	3 (4.8)	0 (0)	2 (2.3)	3 (4.8)	0 (0)	1 (3.2)
CVD family history, *n* (%)	17 (23.9)	18 (34.0)	11 (45.8)	27 (38.0)	13 (25.5)	1 (25)	7 (33.3)
Antihypertensive therapy, *n* (%)	7 (7.6)	24 (38.7)	0 (0)	13 (14.8)	18 (28.6)	1 (16.7)	11 (35.5)
Lipid-lowering therapy, *n* (%)	5 (5.4)	14 (22.9)	2 (7.1)	5 (5.7)	9 (14.3)	1 (16.7)	5 (15.6)
Antiplatelet therapy, *n* (%)	3 (3.3)	6 (9.7)	0 (0)	4 (4.6)	5 (7.9)	0 (0)	5 (16.3)
Glucose (mg/dL)	93.1 ± 8.6	105.8 ± 24.0	94.9 ± 11.4	95.4 ± 8.0	105.8 ± 10.9	97.5 ± 12.6	163.5 ± 50.0
Insulin (U/mL)	5.8 ± 3.2	12.8 ± 5.7	6.5 ± 3.8	7.4 ± 3.4	18.0 ± 8.8	8.9 ± 5.1	15.3 ± 8.2
HOMA-IR	1.4 ± 0.8	3.5 ± 1.8	1.5 ± 0.9	1.7 ± 0.8	4.8 ± 2.5	2.2 ± 1.5	6.1 ± 3.0
Triacylglycerol (mg/dL)	76.4 ± 24.2	118.4 ± 82.7	82.7 ± 24.3	98.8 ± 45.0	175.4 ± 96.1	158.0 ± 71.0	183.4 ± 110.1
Total cholesterol (mg/dL)	216.3 ± 32.6	211.6 ± 43.1	230.7 ± 36.2	226.7 ± 39.5	233.6 ± 49.6	221.3 ± 33.6	207.1 ± 38.9
LDL cholesterol (mg/dL)	139.7 ± 27.9	140.0 ± 33.8	146.1 ± 33.6	150.9 ± 37.8	152.4 ± 46.0	139.5 ± 23.1	129.8 ± 35.2
HDL cholesterol (mg/dL)	61.2 ± 17.6	49.2 ± 14.8	68.0 ± 11.2	55.9 ± 12.9	45.9 ± 10.7	50.3 ± 12.8	41.5 ± 8.4
Urate (mg/dl)	4.7 ± 1.2	5.8 ± 1.3	4.8 ± 1.4	6.0 ± 1.4	6.8 ± 1.2	5.5 ± 1.9	6.4 ± 1.5
ALT, IU/L	13.5 ± 5.8	19.2 ± 8.7	13.3 ± 6.0	21.7 ± 9.3	24.4 ± 12.5	19.0 ± 9.2	29 ± 20
ALP, IU/L	88.9 ± 29.8	94.5 ± 25.9	86.9 ± 26.5	87.0 ± 23.9	95.5 ± 33.9	110.5 ± 25.3	94.0 ± 33.6
GGT, IU/L	16.9 ± 9.8	27.5 ± 20.1	15.9 ± 6.9	26.2 ± 18.9	37.0 ± 29.7	19.2 ± 8.8	39.3 ± 64.8
Creatinine (mg/dl)	0.9 ± 0.2	0.9 ± 0.2	0.9 ± 0.2	1.0 ± 0.2	1.0 ± 0.1	0.9 ± 0.2	1.0 ± 0.2
GFR MDRD (ml/min/1.73 m^2^)	78.6 ± 17.3	86.1 ± 18.1	78.3 ± 16.5	77.5 ± 14.0	78.7 ± 13.1	93.4 ± 14.6	80.2 ± 18.5
GFR CPK-EPI (ml/min/1.73 m^2^)	87.3 ± 17.3	93.2 ± 13.5	87.3 ± 14.6	86.7 ± 14.0	88.5 ± 13.2	91.5 ± 14.3	86.3 ± 16.7
Urine Albumin to creatinine ratio, mg/g	1.3 ± 1.9	1.8 ± 2.2	0.8 ± 0.6	1.8 ± 2.8	1.7 ± 2.1	2.7 ± 2.2	3.6 ± 4.8
BAAT Fibrosis Score (points)	-	-	1 ± 0	2 ± 0	2 ± 1	1 ± 1	3 ± 1
Low likelihood of fibrosis ( ≤ 1); *n* (%)	-	-	28 (100)	12 (13.6)	1 (1.6)	4 (66.7)	0 (0)
Indeterminant score (2–3); *n* (%)	-	-	0	76 (86.4)	61 (96.8)	2 (33.3)	29 (93.5)
Low likelihood of fibrosis (≥4); *n* (%)	-	-	0	0 (0)	1 (1.6)	0 (0)	2 (6.5)

Values are expressed as mean (SD), unless otherwise stated.

*ALT*, alanine aminotransferase; *ALP*, alkaline phosphatase; *BMI*, body mass index; *CUNBAE*, Clínica Universidad de Navarra-body adiposity estimator; *CPK-EPI*, chronic kidney disease epidemiology collaboration equation; *FLD*, fatty liver disease; *GGT*, glutamyl transferase; *GFR*, glomerular filtration rate; *HDL*, high density lipoprotein; *HOMA*, homeostasis model assessment for insulin resistance; *LDL*, low density lipoprotein; *MAFLD*, metabolic dysfunction–associated fatty liver disease; *MD*, metabolic dysfunction; *MDRD*, modification of diet in renal disease equation; *MHO*, metabolically healthy obesity; *MUHO*, metabolically unhealthy obesity; *T2D*, type 2 diabetes.

**Table 3:** Cardiovascular characteristics of patients included in the study.

	**All patients**	**Non- FLD**		**FLD**				
		**Non-FLD nor MD**	**Non-FLD with MD**	**non-MD FLD**	**MAFLD-MHO**	**MAFLD-MUHO**	**MAFLD-MD**	**MAFLD-T2D**
*n*	370	92	62	28	88	63	6	31
VAT (mL)	3659 ± 2058	2239 ± 1675	3933 ± 1770	1505 ± 926	3916 ± 1522	5272 ± 1932	3099 ± 1698	5376 ± 1462
SCAT (mL)	5300 ± 3096	4118 ± 1593	6166 ± 2417	3803.6 ± 1270.7	5310 ± 2204	6278 ± 2423	3816 ± 1129	6702 ± 7619
VAT/SCAT Ratio	0.74 ± 0.39	0.56 ± 0.39	0.70 ± 0.36	0.41 ± 0.25	0.80 ± 0.33	0.89 ± 0.29	0.84 ± 0.44	1.10 ± 0.43
Mean EAT (mL)	158.6 ± 91.2	107.9 ± 75.2	158.9 ± 84.2	94.5 ± 46.4	160.9 ± 69.8	222.7 ± 96.4	143.4 ± 82.8	232.7 ± 90.3
EATi (mL)	81.6 ± 42.5	59.6 ± 36.2	79.8 ± 38.1	55.9 ± 27.3	82.4 ± 33.4	109.3 ± 47.2	78.6 ± 39.9	116.2 ± 39.9
Mean CAC Score (Agatston Score)	73.1 ± 212.9	37.4 ± 121.1	54.3 ± 190.5	25.1 ± 74.4	63.1 ± 167.1	84.5 ± 180.9	27.5 ± 33.8	273.4 ± 486.3

Values are expressed as mean (SD), unless otherwise stated.

*CAC*, coronary artery calcium; *FLD*, fatty liver disease; *MAFLD*, metabolic dysfunction–associated fatty liver disease; *EAT*, epicardial adipose tissue; *EATi*, indexed epicardial adipose tissue; *MD*, metabolic dysfunction; *MHO*, metabolically healthy obesity; *MUHO*, metabolically unhealthy obesity; *SCAT*, subcutaneous adipose tissue; *VAT*, visceral adipose tissue.

### Subclinical cardiovascular disease of the patients included in the study

**Figure 3:**
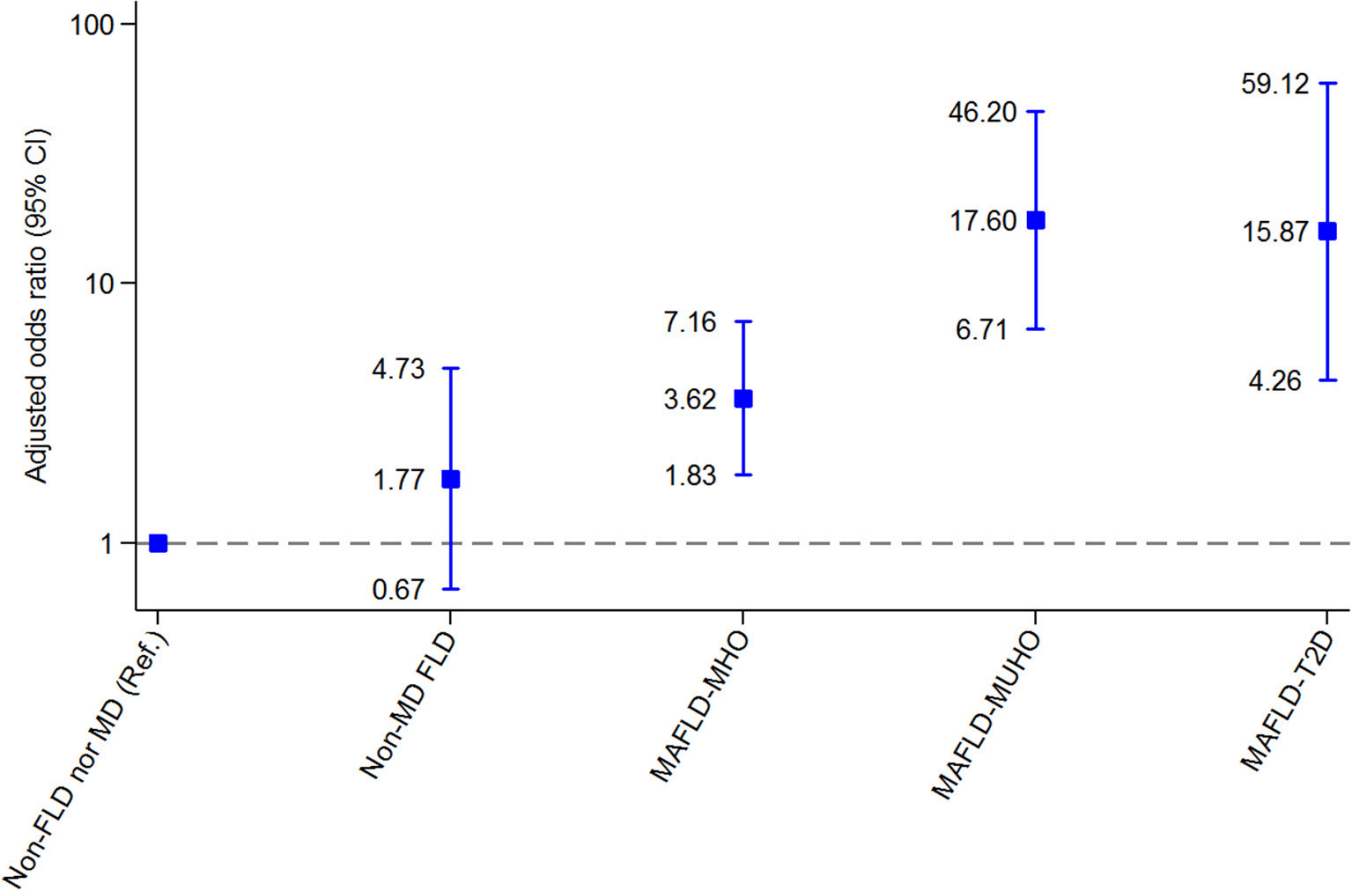
Associations between metabolic phenotypes of fatty liver disease and high indexed epicardial adipose tissue (> 68.1 mL). 95% CI, 95% confidence interval; FLD, fatty liver disease; MAFLD, metabolic dysfunction–associated fatty liver disease; MD, metabolic dysfunction; MHO, metabolically healthy obesity; MUHO, metabolically unhealthy obesity; T2D, type 2 diabetes.

Figure 3 and Supplementary Table 3 present the CVD risk assessed through EATi after adjustment for age and sex. The reference group was defined as patients without FLD nor MD (non-FLD nor MD). Regarding EATi, in multivariate analysis, patients with MAFLD-T2D and MAFLD-MUHO had the highest risk for CVD compared with the reference group (OR 15.87, 95% CI 4.26–59.12 and OR 17.60, 95% CI 6.71–46.20, respectively), patients with MAFLD-MHO were also at risk for CVD (OR 3.62, 95% CI 1.83–7.16), and patients with non-MD FLD did not have a significantly increased risk (OR 1.77; 95% CI, 0.67–4.73).

**Figure 4:**
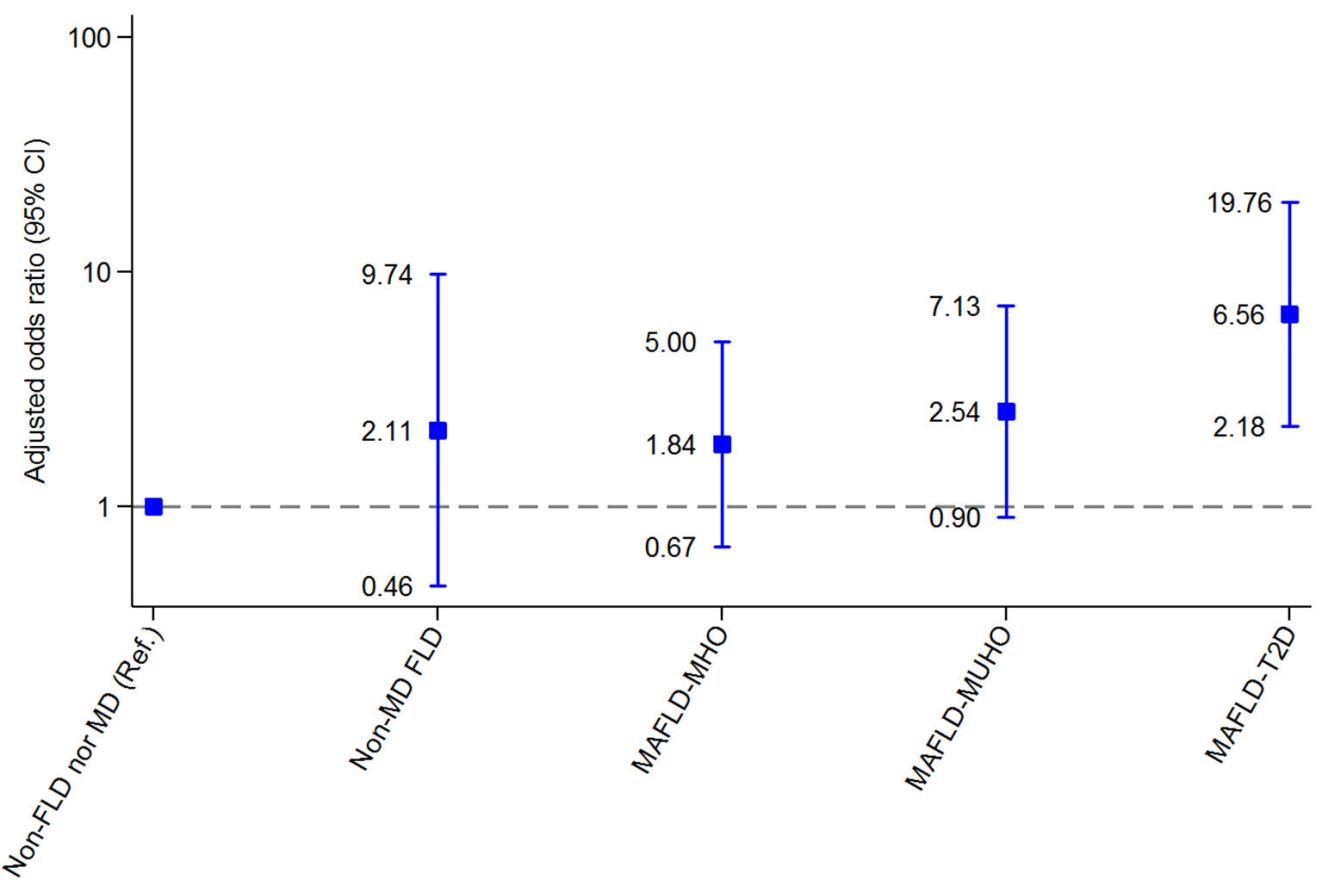
Associations between metabolic phenotypes of fatty liver disease and moderate to severe coronary artery calcification (Agatston CAC score > 100). 95% CI: 95% confidence interval. 95% CI: 95% confidence interval; FLD, fatty liver disease; MAFLD, metabolic dysfunction–associated fatty liver disease; MD, metabolic dysfunction; MHO, metabolically healthy obesity; MUHO, metabolically unhealthy obesity; T2D, type 2 diabetes.

Figure 4 and Supplementary Table 4 present the CVD risk assessed through Agatston after adjustment for age and sex. The reference group was defined as patients without FLD nor MD (non-FLD nor MD). Regarding CAC, in multivariate analysis, patients with MAFLD-T2D had a significant increased risk for CVD compared with the reference group (OR 6.56, 95% CI 2.18–19.76). MAFLD-MUHO, MAFLD-MHO and non-MD FLD did not have significantly increased risk compared with the reference group (OR 2.54, 95% CI 0.90–7.13; OR 1.84, 95% CI 0.67–5.00 and OR 2.11, 95% CI 0.46–9.74, respectively).

In patients with FLD, BAAT fibrosis score significantly correlated with VAT (0.460; *p* < 0.01), VAT/SCAT ratio (0.386; *p* < 0.01), EATi (0.334; *p* < 0.01), and with the presence of CAC (0.235; *p* < 0.01).

## Discussion

Changing from the NAFLD to the MAFLD criteria may help clinicians identifying individuals at higher risk for CVD. Patients with MAFLD–T2D and MAFLD–MUHO had the highest risk for CVD assessed through EATi; nonetheless, patients with MAFLD-MHO were also at risk for CVD. Accordingly, the present study provides novel information for the clinical significance of the different subtypes of MAFLD regarding subclinical CVD and CVD risk in a primary prevention setting. Evidence is needed to widely accept new FLD criteria, thus, some professionals conceive that the proposed MAFLD definition is not flawless and can create unnecessary confusion that could negatively impact research (24).

Regarding cardiovascular morbidity, two studies have investigated CVD outcomes in the different subtypes of patients with MAFLD (4, 14). In both, MAFLD predicted cardiovascular events better than NAFLD. In line with our findings, a higher incidence rate of CVD was found in patients with MAFLD-T2D (4). Interestingly, the patients with “lean” MAFLD had a higher incidence of CVD vs. the patients with MAFLD-OW (465 vs. 307 per 100,000 person-years) (14). We cannot compare our results with this finding, thus, the “lean” MAFLD subgroup may include patients with MD and our cohort included a small number of patients with MAFLD-MD, thereby, we cannot make adequate conclusions in this subgroup. Similarly, although in patients with established CVD, Liu et al. (25) found that patients with MAFLD-T2D had the highest incidence of major adverse cardiac events (MACEs), while MAFLD-OW and MAFLD-MD had similar incidence of MACEs. MAFLD significantly improved the predictive ability of MACEs if added to a model consisting of traditional risk factors improvement in patients with established CVD. In summary, there has been some attempts to characterize the different subtypes of MAFLD; three studies have investigated CVD outcomes of patients with MAFLD, one of them in patients with established CVD. In agreement with previous studies, in the comparison of the different MAFLD subgroups, our data show that in FLD, T2D seems to be the most important driver of CVD, rather than other metabolic abnormalities. A recent meta-analysis found that NAFLD increases the risk of CVD in populations with comparable T2D profiles (26). Moreover, different studies have evidenced higher fibrosis biomarkers in patients with T2D (13, 27). To reduce CVD morbimortality, MAFLD diagnosed through T2D should have an early cardiovascular risk assessment and evaluation of liver fibrosis through non-invasive tests (such as liver elastography and/or liver fibrosis serum biomarkers) (28, 29). It is imperative to provide an intensified control of risk factors through lifestyle intervention favoring weight loss and prescribing antidiabetic drugs with known beneficial effect over NAFLD progression.

To date, evidence consistently refers to MAFLD definition as a practical and convenient term superior to the previous NAFLD definition for identifying patients at high risk for hepatic and extrahepatic complications. Our study attempts to compare the CVD risk of the different MAFLD subtypes but including the different metabolic profiles proposed for overweightness: MHO and MUHO. In line with our findings, several studies have proven CVD risk in both profiles of patients (30). Nonetheless, it has been a matter of debate (31). Our study shows that the term MHO is not a suitable definition and should be avoided, as MHO confers low but relevant subclinical CVD (32), and, with time, patients may have a MUHO, as described by El-L3pez et al. (33). The concentrations of inflammatory cytokines in the different phenotypes of obesity (34, 35), supports the continuum of adipose tissue dysfunction that gradually leads to conversion to an unhealthy phenotype contributing to the development and progression of atherosclerosis (26, 30, 36). Interestingly, MHO and MUHO showed a very similarly altered adipokine and inflammatory profile involved in tissue remodeling in VAT and in the liver (37). In this line, Ampuero et al. (38) found that patients with MUHO had a higher prevalence of NASH compared to MHO, but MHO had a higher prevalence of NASH compared to patients with a healthy metabolically status. Biopsy proven NASH was progressively increased according to the number of metabolic risk factors. Similar data was gathered from the third National Health and Nutrition Examination Surveys 1988–1994 (NHANES III 1988–1994) of the United States (4,087 patients with MAFLD) (13). MAFLD with more metabolic conditions were more likely to have advanced fibrosis (assessed by NAFLD fibrosis score and FIB-4 fibrosis score), even after adjusting for the severity of liver steatosis and alcohol intake. Undoubtfully, metabolic health is a dynamic process at high risk of transition to unhealthy phenotypes.

EATi is considered an earlier and improved subrogate marker of CVD in patients without CAC (39), mediator of cardiac arrhythmias (40) and left ventricular diastolic dysfunction (41). Thus, our findings may be an important addition to prior knowledge and highlight the potential of MAFLD definition in clinical practice as it considers metabolic abnormalities, rather than just BMI. It seems that, for clinicians, MAFLD is a more suitable concept to prevent cardiac burden. However, MAFLD new nomenclature excludes a considerable group of patients with FLD without metabolic abnormalities and apparently lower CVD risk (4, 14, 25). Recently, Semmler et al. (42) found that only 52.1%-69.8% of lean patients with NAFLD fulfilled the novel criteria for MAFLD. We have found that the CVD risk in this subgroup of patients is heterogenous. Therefore, as stated by Younossi et al. (24), the non-homogenous nature of NAFLD might not be fully covered by the new MAFLD criteria, thus, “lean” FLD cannot be considered a metabolically benign condition (43). The heterogenous CVD risk may be explained by the variable nature behind FLD without MD: genetic disorders or susceptibilities, infectious-inflammatory disorders (i.e. hepatitis C, HIV, celiac disease), small intestinal bacterial overgrowth and steatogenic drugs (44). For instance, Wijarnpreecha et al. (45) recently found that homozygous PNPLA3 I148M (rs738409) GG genotype had higher overall mortality after adjusting for multiple metabolic risk factors with a tendency of increased cardiovascular mortality after a follow up of 20 years (*n* = 4814 participants). Interestingly, adiposity may influence the effect of genetic variants on NAFLD. Lin et al. (46) found that the GG genotype was associated with a higher risk of NAFLD in lean patients (OR 6.04), compared with patients with overweightness/obesity. Kim et al. (47) recently found that patients with “lean” NAFLD and a high likelihood of liver fibrosis (assessed through non-invasive serum markers) had a significantly higher CVD risk than those with NAFLD-OW with or without significant liver fibrosis. Therefore, the fibrosis and/or cardiovascular assessment through non-invasive methods in this subgroup of patients -excluded from the MAFLD criteria- could be warranted.

The strength of this study is the employment of different methods to assess CVD risk. Besides, this is the first attempt to describe the CVD overload of the different phenotypes of MAFLD in a European population. All of the studies regarding CVD outcomes in MAFLD has been done in Asian populations, who have significantly less CVD events and genetic variants varies among ethnic groups (48). However, our study has various limitations. First, a single evaluation may not entirely reflect a patient's metabolic status. Second, the use of CT-WBS imaging introduces uncertainty to assessment of hepatic steatosis. Nonetheless, such non-invasive imaging method is recognized for evaluation in international guidelines. Third, the low incidence of patients with MAFLD-MD in our cohort does not allow us to give interpretations regarding this group, nevertheless, our findings highlight the importance of performing longitudinal measures in larger cohorts to analyse prognosis in this subgroup of patients. Fourth, we cannot assess the impact of viral hepatitis on CVD burden because hepatitis virus panel is not routinely performed in the Internal Medicine Check-Up Unit. Likewise, dietary intake and genetic predisposition (i.e. PNPLA3 polymorphism) was not analyzed in our cohort. Fifth, our study is limited by the relatively small sample size. However, the patients included in our work are very well-characterized individuals. Last, our results, are derived from middle-aged Spanish adults, so it should be interpreted with caution when applied to different populations.

In conclusion, patients with MAFLD–T2D and MAFLD–MUHO showed the highest risk for CVD, nonetheless, patients with MAFLD–MHO also had a significant increased risk for CVD. MAFLD new criteria reinforced the importance of identifying metabolic phenotypes in populations, as it may help to identify patients with higher CVD risk and, therefore, offer individualized management to aid primary care clinicians in the task of reducing cardiovascular risk. Clinicians can take advantage of this new definition to facilitate diagnosis and patient education, offer more intensive treatments and appropriate preventive measures in higher risk groups. Nonetheless, omitting a small fraction of individuals with metabolically uncomplicated FLD may leave a considerable number of patients unclassified. Although patients with non-MD FLD are excluded from the MAFLD definition, clinicians should monitor, assess the presence of fibrosis, and consider the presence of genetic abnormalities, inflammatory and infectious disease in this population. Large, prospective, well designed, and longitudinal studies are needed to improve the FLD definition and evaluate non-MD FLD impact on health. Additionally, future research may reveal whether prevention and management of MAFLD can modify CVD risk.

## Data availability statement

The raw data supporting the conclusions of this article will be made available by the authors, without undue reservation.

## Ethics statement

The studies involving human participants were reviewed and approved by Ethics committee of the Clínica Universidad de Navarra (Protocol Number 2019.080). Written informed consent for participation was not required for this study in accordance with the national legislation and the institutional requirements.

## Author contributions

CP, GB, GF, JA, and JE designed the study. CP, FM, and AE performed data collection. CP wrote the first draft of the manuscript. All authors contributed to the article and approved the submitted version.

## Conflict of interest

The authors declare that the research was conducted in the absence of any commercial or financial relationships that could be construed as a potential conflict of interest.

## Publisher's note

All claims expressed in this article are solely those of the authors and do not necessarily represent those of their affiliated organizations, or those of the publisher, the editors and the reviewers. Any product that may be evaluated in this article, or claim that may be made by its manufacturer, is not guaranteed or endorsed by the publisher.
