# Supplementary material for: Cardiometabolic characterization in metabolic dysfunction–associated fatty liver disease
Source: Front Med (Lausanne). 2022 Oct 20;9:1023583. doi: 10.3389/fmed.2022.1023583 (PMC9632176; doi:10.3389/fmed.2022.1023583)
Supplement: Supplementary file 1 [file Table_1.pdf]

Supplementary Table 1. Cardiac function assessed through echocardiography of patients included in the study.

	Non- FLD	FLD	p value
**n**	34	35	-
**Systolic dysfunction; n (%)**	0 (0)	0 (0)	-
**LVEF (%)**	62±6	65±6	0.126
**Left ventricular hypertrophy; n (%)**	11 (32.4)	24 (68.6)	0.003
**Dyastolic dysfunction; n (%)**	22 (64.7)	30 (85.7)	0.043

Values are expressed as mean (SD), unless otherwise stated.

FLD, fatty liver disease; LVEF, left ventricular ejection fraction.

Supplementary Table 2. Adjusted means of metabolic phenotypes of fatty liver disease with visceral adipose tissue and visceral adipose tissue/subcutaneous adipose tissue ratio.

**Metabolic Phenotypes**	**VAT**	**VAT/SCAT ratio**
	**Adjusted mean (95% CI)**	**Adjusted mean (95% CI)**
**Non-FLD nor MD**	2594.39 (2278.45 to 2910.32)	0.67 (0.61 to 0.72)
**Non-FLD with MD**	3899.71 (3528.24 to 4271.17)	0.68 (0.61 to 0.75)
**Non-MD FLD**	2264.19 (1689.97 to 2838.40)	0.63 (0.52 to 0.74)
**MAFLD-MHO**	3752.19 (3439.47 to 4064.90)	0.75 (0.69 to 0.81)
**MAFLD-MUHO**	4931.09 (4556.12 to 5306.05)	0.79 (0.72 to 0.86)
**MAFLD-T2D**	4858.80 (4324.18 to 5393.41)	0.96 (0.86 to 1.06)

95% CI, 95% confidence interval; FLD, fatty liver disease; MAFLD, metabolic dysfunction–associated fatty liver disease; MD, metabolic dysfunction; MHO, metabolically healthy obesity; MUHO, metabolically unhealthy obesity; T2D, type 2 diabetes; VAT, visceral adipose tissue; VAT/SCAT ratio, visceral adipose tissue/subcutaneous adipose tissue ratio.

Supplementary Table 3. Associations between metabolic phenotypes of fatty liver disease and high indexed epicardial adipose tissue (>68.1 mL).

**Metabolic Phenotypes**	**High indexed epicardial adipose tissue (>68.1)**	
	**OR (95% CI)**	**P value**
**Non-FLD nor MD**	Reference	Reference
**Non-MD FLD**	1.77 (0.67 to 4.73)	0.251
**MAFLD-MHO**	3.62 (1.83 to 7.16)	<0.001
**MAFLD-MUHO**	17.60 (6.71 to 46.20)	<0.001
**MAFLD-T2D**	15.87 (4.26 to 59.12)	<0.001

95% CI, 95% confidence interval; FLD, fatty liver disease; MAFLD, metabolic dysfunction–associated fatty liver disease; MD, metabolic dysfunction; MHO, metabolically healthy obesity; MUHO, metabolically unhealthy obesity; OR, odds ratio; T2D, type 2 diabetes.

Supplementary Table 4. Associations between metabolic phenotypes of fatty liver disease and moderate to severe coronary artery calcification (Agatston CAC score>100).

Metabolic Phenotypes	Moderate to severe coronary artery calcification (Agatston CAC score>100)	
	**OR (95% CI)**	**p value**
**Non-FLD nor MD**	Reference	Reference
**Non-MD FLD**	2.11 (0.46 to 9.74)	0.341
**MAFLD-MHO**	1.84 (0.67 to 5.00)	0.235
**MAFLD-MUHO**	2.54 (0.90 to 7.13)	0.077
**MAFLD-T2D**	6.56 (2.18 to 19.76)	0.001

95% CI, 95% confidence interval; FLD, fatty liver disease; MAFLD, metabolic dysfunction–associated fatty liver disease; MD, metabolic dysfunction; MHO, metabolically healthy obesity; MUHO, metabolically unhealthy obesity; OR, odds ratio; T2D, type 2 diabetes.
